# *Toxoplasma gondii* in edible fishes captured in the Mediterranean basin

**DOI:** 10.1111/zph.12630

**Published:** 2019-07-06

**Authors:** Anna Maria Fausta Marino, Renato Paolo Giunta, Antonio Salvaggio, Annamaria Castello, Tiziana Alfonzetti, Antonio Barbagallo, Alessandra Aparo, Fabrizio Scalzo, Stefano Reale, Wilma Buffolano, Maurizio Percipalle

**Affiliations:** ^1^ National Reference Centre for Toxoplasmosis Istituto Zooprofilattico Sperimentale della Sicilia Catania Italy; ^2^ Perinatal Infection Register Campania Region Naples Italy

**Keywords:** fish, food, *Toxoplasma gondii*

## Abstract

The issue of whether market fish can be involved in the transmission of *Toxoplasma gondii* in the marine environment is highly debated since toxoplasmosis has been diagnosed frequently in cetaceans stranded along the Mediterranean coastlines in recent times. To support the hypothesis that fishes can harbour and effectively transmit the parasite to top‐of‐the‐food‐chain marine organisms and to human consumers of fishery products, a total of 1,293 fishes from 17 species obtained from wholesale and local fish markets were examined for *T. gondii* DNA. Real‐time PCR was performed in samples obtained by separately pooling intestines, gills and skin/muscles collected from each fish species. Thirty‐two out of 147 pooled samples from 12 different fish species were found contaminated with *T. gondii* DNA that was detected in 16 samples of skin/muscle and in 11 samples of both intestine and gills. Quantitative analysis of amplified DNA performed by both real‐time PCR and digital PCR (dPCR) confirmed that positive fish samples were contaminated with *Toxoplasma* genomic DNA to an extent of 6.10 × 10^−2^ to 2.77 × 10^4^ copies/ml (quantitative PCR) and of 1 to 5.7 × 10^4^ copies/ml (dPCR). Fishes are not considered competent biological hosts for *T. gondii*; nonetheless, they can be contaminated with *T. gondii* oocysts flowing via freshwater run‐offs (untreated sewage discharges, soil flooding) into the marine environment, thus acting as mechanical carriers. Although the detection of viable and infective *T. gondii* oocysts was not the objective of this investigation, the results here reported suggest that fish species sold for human consumption can be accidentally involved in the transmission route of the parasite in the marine environment and that the risk of foodborne transmission of toxoplasmosis to fish consumers should be further investigated.

**Impacts**

- *Toxoplasma gondii* is an ubiquitous parasite isolated from cetaceans with meningoencephalitis stranded in the Mediterranean basin.
- Extracellular polymeric substances (EPS), surface‐scraping mollusks, shellfish and fish are potentially involved in the transmission pathways of *T. gondii* in the marine environment.
- Market fish contaminated with *T. gondii* could be of a public health concern for workers involved on fish manipulation and consumers besides a marker of environmental pollution.

## 1 INTRODUCTION

The apicomplexan *Toxoplasma gondii *is a global zoonotic parasite affecting humans and animals, both terrestrial and aquatic, that causes a cosmopolitan food and waterborne infection, with an estimated 1–2 billion (approximately 30%) of the world's population infected (Bahia‐Oliveira, Gomez‐Marin, & Shapiro, 2017). For successful transmission, the parasite relies on an heteroxenous life cycle involving an intermediate host, virtually all warm‐blooded animals, wherein the parasite multiplies by asexual reproduction leading to intracellular cysts developing in muscles and other organs and a definitive host (wild and domestic felids) in which sexual reproduction results in the shedding through faeces of countless infective oocysts in the environment. As a result, the risk factors for human and animal infection include consuming infected raw or undercooked meat; ingestion of contaminated water, soil, vegetables or anything contaminated with oocysts shed in faeces; blood transfusion or organ transplants; intrauterine or transplacental transmission; and drinking infected unpasteurized milk (Aguirre et al., 2019).

In the past, oocysts‐acquired infections have been deemed as less important than foodborne infections although they are considered more severe clinically in intermediate hosts than those related to the ingestion of tissue cysts (Hill & Dubey, 2002). Despite the consumption of raw or undercooked meat harbouring *T. gondii* cysts is still considered the most likely source of infection, in the last 40 years' toxoplasmosis outbreaks associated with exposure to oocysts‐contaminated water have been reported in Panama (Benenson, Takafuji, Lemon, Greenup, & Sulzer, 1982), in British Columbia (Bowie et al., 1997), in Brazil (De Moura et al., 2006) and in India (Balasundaram, Andavar, Palaniswamy, & Venkatapathy, 2010) with the latter two episodes resulting from the ingestion of municipal drinking water. Furthermore, the majority (78%) of congenital toxoplasmosis cases from four epidemics in North America originated from oocysts exposure, though only 49% of these cases could be confirmed as foodborne (Aguirre et al., 2019).

The substantial improvements made in animal husbandry biosecurity measures as well as the enforcement of good manufacturing practices in the meat industry has led to the reduction of the risk of meat‐based transmission of the infection and shifted the attention on the role played by *T. gondii* oocysts shed in the environment in the epidemiology of toxoplasmosis. Environmental exposure to *T. gondii* oocysts (Conrad et al., 2005) is acknowledged as an important route for human infection as demonstrated by the steady level of toxoplasmosis prevalence in the human population in the United States (Jones, Kruszon‐Moran, & Wilson, 2003) despite the enforcement of animal health and food safety measures resulting in the decrease of the infection prevalence in market pigs which are unanimously regarded as the main foodborne source of human infection. Moreover, long‐term observational studies carried out on congenital toxoplasmosis transmitted by infected pregnant women showed that infection could not always be linked to typical risk factors, such as consumption of cysts‐contaminated meat, involved with toxoplasmosis and that a different route needed to be sought. Finally, the development of more sensitive analytical methods for the detection of oocysts in soil and water samples has added further evidences of the spread of oocysts shed in the environment whose role is no longer seen as secondary to tissue cysts‐based transmission.

Environmental pollution with *T. gondii* oocysts can be accounted for the contamination of both freshwater and marine aquatic environments where *Toxoplasma* infection is being frequently diagnosed in several sea mammals (Fayer, Dubey, & Lindsay, 2004) that are at the top of the aquatic food chain. Field surveys suggest that as many as eight different families of marine mammals are susceptible to *T. gondii* infection (Dabritz et al., 2007) including the endangered southern sea otter (*Enhydra lutris nereis*) in which toxoplasmosis is responsible for high mortality and slow rate of population recovery (Conrad et al., 2005). Cetaceans are also susceptible to *T. gondii* infection that is often found in individuals stranded in the Mediterranean coastlines due to infection with viral and bacterial pathogens (*Morbillivirus*, *Herpesvirus* and *Brucella* spp) (Di Guardo & Mazzariol, 2016) with toxoplasmosis more likely to be secondary to concurrent immunosuppression.

Anthropogenic coastline pollution with sewage or surface run‐off of freshwater contaminated with the *T. gondii* oocysts is likely the key factor in the epidemiology of toxoplasmosis in the marine environment with feral and domestic cats playing as the sole source of oocysts. Following infection, a single cat can shed millions of oocysts within 1 week. Also cats' defecation behaviour, such as faeces burial in shady areas, increases the survival of oocysts that can persist in the environment longer than 1 year. Flood‐related natural events could also drive the transmission of terrestrial pathogens like *T. gondii* especially in estuarine environments.

Many gaps remain to be filled in the transmission pathways and pathogenesis of toxoplasmosis in aquatic mammals. For those cetaceans exclusively from offshore waters, contact with oocysts‐contaminated wastewaters discharged from ships has been advocated (Di Guardo et al., 2010). Recently novel routes of oocysts transmission involving a complex interaction of suspended bioparticles, biofilms, small invertebrates and gastropods have been hypothesized whereas mammals can also become infected nearby the coast prior to stranding or via consumption of fishes or mussels carrying the parasite.

In the search for potential hosts involved in the transmission of the parasite between terrestrial and marine environments where it is known to trigger the infection in competent hosts like sea mammals, we searched for *T. gondii* DNA in fishes belonging to small to medium‐size species positioned at different levels of the aquatic food webs. The main goal of the study was to assess whether contaminated fishery products could be a further risk factor of foodborne toxoplasmosis for human consumers and also an occupational hazard for fishing and fish processing operators considering that the fish species tested are among those most commonly harvested and sold for human consumption in the Mediterranean area.

## 2 MATERIALS AND METHODS

Sampling was carried out from June 2017 to April 2018 at MAAS (Sicily Agro‐Food Markets) the largest wholesale market for fruit, vegetable, flower and fish in Sicily and in several local small‐size fisheries where fishes captured in the FAO fishing area 37.2.2 are mostly traded. On a bi‐monthly basis, fishes were chosen randomly among those commonly found in fish markets according to fishing season and purchased by local population for fresh consumption. Approximately two hundred units from different fish species per sampling session were collected randomly from retail boxes and kept refrigerated until processing by using cold packs in thermally insulated containers. For the purpose of the study, only fishes caught in the previous 24 hr were collected and processed within the subsequent 24 hr.

An overall total number of 1,293 fish units from 17 different species were sampled. The number of collected fishes per species ranged from 350 units for European anchovy (*Engraulis encrasicolus*) to a minimum of a single unit for the European conger (*Conger conger*), the thornback ray (*Raya clavata*) and the cow bream (*Sarpa salpa*). Since the main goal of the study was to evaluate the extent to which *T. gondii* in fish can be a food safety issue under everyday life habits of relatively small quantities of fresh fish purchased for consumption, we decided to include in the survey also fish species for which few units were collected because of limited availability in markets at the time of sampling. Detailed description of sampling is shown in Table 1.

**Table 1:** Fish specimens collected from fish markets

Taxonomic name	Common name	Number of fish units sampled	Number of pooled samples per tissue
*Argentina sphyraena*	Argentine	6	1
*Arnoglossus laterna*	Mediterranean scaldfish	6	1
*Boops boops*	Bogue	260	26
*Conger conger*	European conger	1	1
*Diplodus sargus*	White seabream	18	3
*Engraulis encrasicolus*	European anchovy	350	35
*Merluccius merluccius*	European hake	90	15
*Mullus barbatus*	Red mullet	110	11
*Pagellus acarne*	Axillary seabream	80	8
*Pagellus erythrinus*	Common pandora	18	3
*Raja clavata*	Thornback ray	1	1
*Sardina pilchardus*	European pilchard	200	20
*Sarpa salpa*	Cow bream	1	1
*Scorpaena scrofa*	Red scorpionfish	3	1
*Serranus cabrilla*	Comber	5	1
*Spicara maena*	Blotched picarel	24	4
*Trachurus trachurus*	Atlantic horse mackerel	120	15
Total		1,293	147

In order to process and analyse samples of similar size, fishes were not individually tested but each species collected from the market was divided in groups of 3, 5, 6, 8 or 10 units according to fish size and weight. From each fish, intestine, gills and multiple aliquots of skin–skeletal muscles complex (to a maximum weight of 10 g) were split in asepsis, separately. Guts were examined to determine the fishes' capacity to retain oocysts as it was previously documented (Massie, Ware, Villegas, & Black, 2010). Likewise, gills were analysed to determine whether they can trap oocysts filtered from seawater. Skin and muscle were not separated prior to analysis as it was considered unrealistic to avoid potential DNA cross‐contamination from specimen to specimen contact during processing and also to evaluate the extent to which ingestion of skin and meat from potentially *T. gondii* contaminated fish may pose a risk of infection for consumers.

Single tissue types of each group were pooled separately, transferred into sterile stomacher bags and blended with a mortar. The homogenate was diluted with an equal volume of TE (Tris‐EDTA) buffer and re‐homogenized with a stomacher. In conclusion, *T. gondii* DNA testing was performed on overall total number of 1,293 fish units pooled in 147 groups.

DNA extraction was performed on a volume of 200 µl of homogenate using the nexttec^™^^1‐Step^ DNA Isolation Systems kit (Nexttec) according to the manufacturer's instructions. DNA was quantified by NanoDrop® ND‐1000 spectrophotometer.

A real‐time PCR method targeting the 529‐bp repeated fragment of *T. gondii* genome (GenBank accession no. AF146527) was applied on sampled tissues (Marino et al., 2017). PCR was performed in a 20 μl final volume containing 10 μl of master mix 2×, added to nuclease‐free water (4 μl), 10 pmol of each primer AF1 (CACAGAAGGGACAGAAGT) and primer AF2 (TCGCCTTCATCTACAGTC), 5 pmol of the labelled TaqMan probe AF 529 (6FAM‐CTCTCCTCCAAGACGGCTGG‐ BHQ), 2 µl of 10× Exo IPC‐VIC (Applied Biosystems), 0.5 µl of 50× Exo IPC DNA (Applied Biosystems) and 40 ng of DNA template. The amplification protocol was performed as follows: 2 min incubation at 50°C, a first step denaturation at 95°C for 10 min and 40 cycles of a 2‐step amplification (denaturation at 95°C for 15 s, annealing and extension at 60°C for 60 s). Each batch of PCR assays included *T. gondii* genomic DNA (ATCC 50174D™ from Rh‐88 *T. gondii*; LGC Standards) as positive control template and molecular grade water as negative control template.

Quantification of detected DNA from positive samples was subsequently assessed by a laboratory‐developed quantitative PCR (qPCR) in order to estimate the parasitic load. qPCR was performed as follows: Serial dilution of the Standard DNA (ATCC 50174D™ gDNA from Rh‐88 *T. gondii*) from 10^7^ to 10^3^ copies/ml were prepared, diluting the standard DNA in molecular grade water. For each sample, the reaction mixture was prepared by mixing 10 µl of TaqMan Genotyping Master Mix (Applied Biosystems), 0.5 µl each of forward and reverse primer at the initial concentration of 20 pmol/µl, 0.5 µl of FAM‐probe at the initial concentration of 10 pmol/µl, 2 µl of 10× Exo IPC‐VIC (Applied Biosystems), 0.5 µl of 50× Exo IPC DNA (Applied Biosystems) and 4 µl molecular graded water. Eighteen microliter of mix was aliquoted to the corresponding wells of a PCR microplate. Two microliter of standard dilutions and 2 µl of total DNA at the initial concentration of 20 ng/µl were then added to the corresponding wells. The amplification programme consisted of one cycle at 50°C for 2 min, 1 cycle at 95°C for 10 min and then 40 cycles of a 2‐step amplification: denaturation at 95°C for 15 s, annealing and extension at 60°C for 60 s. The fluorescence signal was acquired at step 4.

Each qPCR assay was run in triplicate, and the average value was kept as a measure of DNA concentration. Using the standard curve as a reference, the real‐time PCR thermocycler's dedicated software calculated the amount of *T. gondii* DNA contained in each well, expressed as number of copies of the target DNA (529 bp repeat element dispersed in *T. gondii* genome) sequence/ml.

According to validation performed in compliance with OIE and Codex Alimentarius guidelines (Marino et al., 2017), the overall sensitivity and specificity of both applied qualitative and qPCR methods was 100%. The limit of detection threshold was 0.01 pg/μl.

As a further step for the quantification of *T. gondii* DNA, a digital PCR (dPCR) protocol was employed. A notable benefit of dPCR over qPCR is that this analysis technique requires no standard, calibration or information about the molecular weight distribution of the template molecules (Sanders, Mason, Foy, & Huggett, 2013). A laboratory‐developed TaqMan Real‐time protocol with QuantStudio™ 3D Digital PCR Master Mix (Thermo Fisher Scientific) was employed according to the manufacturer's instruction with specific primers and TaqMan probe AF 529 (6FAM‐CTCTCCTCCAAGACGGCTGG‐ BHQ).

The sample mix was added on each chip and loaded on ProFlex™ 2× Flat PCR System with the following programme: a first step denaturation at 96°C for 10 min and 40 cycles of a two‐steps amplification (denaturation at 98°C for 30 s, annealing and extension at 60°C for 60 s). Absolute quantification was determined using QuantStudio 3D Digital PCR System (Thermo Fisher Scientific) and analysed with QuantStudio 3D Analysis Suite Cloud Software (Thermo Fisher Scientific).

## 3 RESULTS

*Toxoplasma gondii* contaminating DNA was found in 12 of 17 fish species tested with overall 32 positive samples out of 147. The largest number of positive samples was found in *Boops boops* with *T. gondii* DNA being detected in 6/26 pooled skin/muscle, 4/26 pooled gills and 3/26 pooled intestine samples. Positive species also included the following: *Trachurus trachurus* (4/15 skin/muscle samples); *Engraulis encrasicolus* (2/35 gills and 1/35 intestine samples); *Mullus barbatus* (3/11 intestine samples); *Pagellus acarne* (2/8 gills, 1/8 intestine and 1/8 skin/muscle samples); *Pagellus erythrinus* (1/3 gills, 2/3 intestine and 1/3 skin/muscle samples); *Merluccius merluccius* (1/15 gills and 1/15 skin/muscle samples); *Arnoglossus laterna* (1/1 gills sample); *Diplodus sargus* (1/3 skin/muscle sample); *Raja clavata* (1/1 skin/muscle sample); *Scorpaena scrofa* (1/1 intestine sample); *Spicara maena* (1/4 skin/muscle sample). Skin and muscles samples resulted the most contaminated with *T. gondii* DNA (16/147 samples) with slightly lower values for gills and intestine samples (11/147 samples; Table 2). Only in one group of *B. boops T. gondii* DNA was detected in all types of tissues examined.

**Table 2:** Results of PCR analysis on samples of pooled fish tissues

Species	No. of samples per tissue	No. of samples positive for *Toxoplasma gondii*/total		
		Gills	Intestine	Skin/muscle
*Argentina sphyraena*	1	0/1	0/1	0/1
*Arnoglossus laterna*	1	1/1	0/1	0/1
*Boops boops*	26	4/26	3/26	6/26
*Conger conger*	1	0/1	0/1	0/1
*Diplodus sargus*	3	0/3	0/3	1/3
*Engraulis encrasicolus*	35	2/35	1/35	0/35
*Merluccius merluccius*	15	1/15	0/15	1/15
*Mullus barbatus*	11	0/11	3/11	0/11
*Pagellus acarne*	8	2/8	1/8	1/8
*Pagellus erythrinus*	3	1/3	2/3	1/3
*Raja clavata*	1	0/1	0/1	1/1
*Sardina pilchardus*	20	0/20	0/20	0/20
*Sarpa salpa*	1	0/1	0/1	0/1
*Scorpaena scrofa*	1	0/1	1/1	0/1
*Serranus cabrilla*	1	0/1	0/1	0/1
*Spicara maena*	4	0/4	0/4	1/4
*Trachurus trachurus*	15	0/15	0/15	4/15
Total	147	11	11	16

Quantification of amplified DNA by both quantitative real‐time PCR and dPCR confirmed that positive fish samples were contaminated with *Toxoplasma* genomic DNA to an extent of 6.10 × 10^−2^ to 2.77 × 10^4^ copies/ml (qPCR) and of 1–5.7 × 10^4^ copies/ml (dPCR). The estimated heaviest parasitic load was found in pooled samples of *B. boops* intestines (qPCR) and in gills samples of *M. merluccius* (dPCR). Detailed results of quantitative analyses are reported in Table 3.

**Table 3:** Quantitative PCR and digital PCR analysis of *Toxoplasma gondii* DNA detected in positive fish pooled samples

Fish species	No. of positive DNA	Quantitative PCR (copies/ml)			Digital PCR (copies/ml)		
		Gills	Intestine	Skin/muscle	Gills	Intestine	Skin/muscle
Mediterranean scaldfish *(Arnoglossus laterna)*	1	1.69			3.24 × 10^2^		
Bogue (*Boops boops*)	13	3.31	2.77 × 10^4^	4.68	1.8 × 10	1.54 × 10^3^	2.61 × 10
		2.71	1.8 × 10^4^	1.1	3.3 × 10^2^	1 × 10^3^	1.19
		3.4	2.54 × 10^3^	4.41	1.16 × 10^2^	1.41 × 10^2^	2.46 × 10
		2.81		3.57 × 10^2^	2.1		2.61 × 10^2^
				6.91			3.8 × 10
				1.77 × 10^3^			9.8 × 10^2^
White seabream *(Diplodus sargus)*	1			1.15 × 10			NP
European anchovy (*Engraulis encrasicolus)*	3	3.05	2.4 × 10^2^		NP	NP	
		1.91			NP		
European hake *(Merluccius merluccius)*	2	1.53 × 10^4^		1.81 × 10	5.70 × 10^4^		NP
Red mullet (*Mullus barbatus*)	3		6.10 × 10^−2^			1.00	
			3.58			1.20	
			1.95			1	
Axillary seabream (*Pagellus acarne*)	4	2.75	9.38	1.06 × 10^3^	1.25 × 10^2^	1 × 10	3.94 × 10^3^
		4.65			2.16 × 10^2^		
Common pandora (*Pagellus erythrinus*)	4	3.35	1.20	1.18 × 10	1.57 × 10^2^	5.90 × 10	5.6 × 10^2^
			9.6 × 10^−1^			3.92 × 10	
Thornback ray (*Raja clavata)*	1			3.77 × 10			2.70 × 10^2^
Red scorpionfish (*Scorpaena scrofa*)	1		1.76 × 10^2^			1.5 × 10^3^	
Blotched picarel (*Spicara maena*)	1			4.41			6.40 × 10
Atlantic horse mackerel (*Trachurus trachurus)*	4			2.67		2.60 × 10	
				7.06 × 10^−1^		3.30 × 10	
				1.7		1.76 × 10	
				6.5 × 10^−1^		2.94 × 10	

Note

NP, not performed due to paucity of DNA.

## 4 DISCUSSION

Fishes found positive in this survey belong to species rather heterogeneous as regards living and dietary/foraging habits. *Toxoplasma gondii* DNA was detected equally in mainly piscivorous species (*A. laterna*, *M. merluccius*, *R. clavata, S. scrofa and T. trachurus*), in omnivorous fishes (*B. boops, P. acarne and P. erythrinus*) and in species feeding on small benthic invertebrates or zooplankton (*D. sargus, M. barbatus, Sp. maena and E. encrasicolus*). Most of these species are demersal or benthopelagic living on different type of seabeds (sand, mud, rocks or seagrass bed) where *T. gondii* oocysts are more likely to settle aided by aquatic invertebrates that enhance settling and help benthos concentration (Aguirre et al., 2019). With this respect, albeit accidental, fish tissue contamination with *T. gondii* could be land‐based and detectable in fishes captured shortly after exposure. Also, some of the species included in the study, like the European anchovies, are migratory and gregarious fishes dwelling in large banks nearshore during the reproduction season at the beginning of spring moving away into deeper waters at the end of summer and in autumn (Patti et al., 2011) hence supporting the hypothesis of an open‐sea transmission cycle of *T. gondii* that could affect larger predators and sea mammals. Anchovies feed by filtering food particles in water and likewise mussels can capture oocysts disperse in the water or attached to marine aggregates and retain them temporarily in the digestive tract or in the gills. Finding of *Toxoplasma* DNA in skin samples could also result from cross‐contamination between fishes when gut content is released following capture stress or manipulation.

Our findings that fishery products are somehow exposed to *T. gondii* are consistent with reports suggesting that *T. gondii* can survive in the sea (Arkush et al., 2003; Lindsay et al., 2003, 2004) and may affect many aquatic species ranging from small invertebrates to market seafood and fishes and mammals at the top of the aquatic chain. Investigations have demonstrated that sporulated *T. gondii* oocysts can remain viable when stored at 4°C for 24 months in seawater (Lindsay & Dubey, 2009). Seafood like mussels (Arkush et al., 2003) and oysters (Lindsay et al., 2001) have also been recognized as capable of removing *T. gondii* oocysts from seawater.

Further insights on the epidemiology of *T. gondii* in marine waters come from recent studies of extracellular polymeric substances (EPS) (Shapiro et al., 2014). These substances are “relatively abundant”—although subject to seasonal changes depending upon microbiota blooming—in the sea environment and play a number of biophysical roles in promoting the aggregation of bioparticles and providing the structural matrix of biofilms. Due to their sticky properties, EPS can mediate transportation of soil‐derived pathogens, including *T. gondii*, in the marine ecosystem either by incorporating into marine macroaggregates (e.g., marine snow) or through direct adhesion to biofilms coated on the surface of seaweeds or other benthic organisms (Wotton, 2004). Both mechanisms are likely to increase the chance of pathogens entry into the marine food web through aggregate‐consuming invertebrates such as bivalves or surface‐scraping molluscs such as snails (Shapiro et al., 2014) with infection of higher‐level organisms (sea mammals) occurring subsequently. Gastropods can then be considered a major source of infection since they represent a large component of the marine environment. Under experimental conditions, surrogates of *T. gondii* oocysts were detected attached in kelp (*Macrocystis pyrifera*) blades when EPS substances form the matrix of the biofilm covering the blades' surface (Mazzillo, Shapiro, & Silver, 2013). Similarly, EPS attached to sediment particles have also been proved to be ingested by deposit‐feeding marine organisms (Hoskins, Stancyk, & Decho, 2003). These studies provide a novel transmission route for *T. gondii* in the marine environment and could explain why gross pathology signs of toxoplasmosis have been detected not only in coastal species like the bottlenose dolphin (*Tursiops truncates*), that can get infected through faecal oocyst contamination flowing from land to sea, but also in typically offshore‐living dolphin species like the striped dolphin (*Stenella coeruloalba*) (Di Guardo & Mazzariol, 2013).

Fishes are not considered competent biological hosts for *T. gondii* although experimental studies indicate that common commercial species like anchovies and sardines are able to pass *T. gondii* oocyst through their intestine following exposure to oocysts‐spiked sea water (Massie et al., 2010).

Strictly speaking of toxoplasmosis, only in vitro infection has been documented for fishes (Omata et al., 2005; Sanders et al., 2015). Following intraperitoneal inoculation of *T. gondii* bradyzoites in zebrafish (*Danio rerio*), tachyzoites were detected through histological examination in several tissues including skeletal muscle (Sanders et al., 2015). A similar study carried out in goldfishes (*Carassius auratus*) showed that *T. gondii* tachyzoites can persist up to 3 days post‐infection (intramuscular inoculation) as evidenced by PCR analysis of inoculated tissues and by bioassay in mice intraperitoneally inoculated with tissue homogenate from experimentally infected fishes (Omata et al., 2005). Also in vitro infection of oviduct epithelial cells of goldfish showed active intracellular reproduction of *T. gondii* tachyzoites within 6 hr of inoculation (Omata et al., 2005). However, a temperature of 37°C was an absolute limiting factor of the in vitro studies, since at lower temperatures *T. gondii* tachyzoites do not appear to be able to penetrate and multiply in the somatic host cells (Omata et al., 2005; Sanders et al., 2015). Therefore, the existence of a *T. gondii* biological cycle in fish similar to that occurring in mammals is highly unlikely and consequently trophically transmitted infection in marine mammals should be incidental and limited to the predation of sea organisms mechanically carrying oocysts.

The issue of the viability and availability of oocysts dispersed in the marine environment is also highly debated and whether oocysts‐carrying fishes are able to mechanically transmit viable parasites to larger predators at the top level of the food chain, including human consumers, remains controversial. Oocyst viability, assessed through bioassay or molecular diagnosis of toxoplasmosis on spf mice fed with infected tissues from sardines exposed to *T. gondii* oocysts, was confirmed for fishes exposed to high parasite load (at least 100,000 oocysts/L of seawater) (Massie et al., 2010). Additionally filter‐feeding fishes exposed to oocysts for 2 hr are able to retain oocysts in the alimentary canal for at least 8 hr (anchovies) although oocysts viability has been proven only for contaminated sardine tissues (Massie et al., 2010). As filter feeders specialized in eating microparticles (Kucas, 1986), both anchovies and sardines are naturally suited to consume *T. gondii* oocysts as they enter the nearshore environment; as the fishes migrate offshore, they could then serve as biotic vectors for *T. gondii* into the greater marine environment (Massie et al., 2010).

The small pelagic fish species investigated in this study are predominantly caught by purse seiners with or without fish aggregating devices. The EU Council Regulation (EC) No 1967/2006 forbids purse seines within 300 m of the coast or within the 50 m isobath where that depth is reached at a shorter distance from the coast (European Council, 2006). In our opinion, fish contamination with land‐based *T. gondii* oocysts may still occur outside and around these prescribed distances/depths from the coastline. At this distance pollution of the fishing grounds could be due to unauthorized untreated sewage discharged directly into the sea or via freshwater streams, which is unfortunately a known issue in the Mediterranean area, or as the result of heavy run‐offs from severe flooding that are being reported more and more frequently in recent times. Both phenomena could drive the flow of *T. gondii* from land to sea with fishes acting mainly as carriers of oocysts on the body surface or transiently in the guts.

Of course, contamination of surface run‐offs with oocysts shed by cats remains the key point to be addressed. Unfortunately, no information is available about the size of the feral and stray cats population in the countries bordering the Mediterranean sea, either about the successful enforcement of capture‐and‐release‐neutering programmes that represents the most effective strategy to control the feline population and reduce the scale of soil and groundwater contamination with the parasite oocysts. With this regard, commitments to law to control the number of feral domestic cats on the landscape to minimize the risk of transmission of *T. gondii* is acknowledged as a part of the local capacity in a One Health approach to address the issue (Aguirre et al., 2019).

On‐board contamination of the fish analysed could not also be ruled out. Fishing vessels are normally equipped with all the necessary facilities for handling, washing, icing and stowing the catch in fish holds. On‐board flake ice‐making facilities are supplied with potable or clean water as prescribed by EU regulations (European Commission, 2004) thus contact of the fishery products with oocysts‐contaminated clean but non‐potable water, during washing or storing in ice, is possible albeit unlikely. Cats presence can be an additional risk factor since it is not rare for cats to board docked boats for feeding on fish waste after the fish is landed. Accurate cleaning of vessels decks and fish holds after processing operations, as well as improving on‐board biosecurity measures to keep off cats from working surfaces and water tanks, should be pursued. Fish workers should also be trained to follow proper hygiene practices and required to wear protective clothing and boots that must be cleaned regularly in order to minimize the risk of fish contamination with environmental *T. gondii* oocysts.

The risk of *T. gondii* occurrence in fishes should therefore be taken into account also as an occupational issue. Operators working in the fishing industry should be aware of the risk of exposure to the parasite both in the primary production stages (fishing and harvesting) and during raw fish handling for the production of processed fishery products like salt cured anchovies. This issue should be addressed especially for female workers that make up the largest part of employees in small enterprises in the south Mediterranean countries where fish processing largely relies on manual labour. Good manufacturing practices must therefore be implemented to reduce the risk for exposed workers namely for pregnant women.

From a food safety perspective, the species that were found to be contaminated with *T. gondii* DNA account for 23.1% of the total fishes caught in Sicily in 2015 (8,355 out of 21,291 tons) (Istituto Nazionale di Statistica [ISTAT], 2015). Moreover, some of the investigated fish species (such as anchovies) are occasionally eaten raw or following curing (marinade) in lemon juice (pH 2) for a few hours. This procedure does not ensure food security since it has been documented that oocysts can retain viability in aqueous 2% sulphuric acid for several years at 4°C (Dumètre & Dardé, 2003; Dumètre et al., 2008).

Although there are no documented evidences of *T. gondii* infection through the consumption of fresh raw or undercooked fish, findings of this study suggest that fishes should be regarded as liable of accidental contamination with the parasite and therefore deemed as a potential source of infection for marine mammals and humans alongside shellfish that are known to be able to capture and concentrate *T. gondii* oocyst from sea water (Aksoy et al., 2014; Esmerini, Gennari, & Pena, 2010). Hence, the guidelines suggested for the safe storing, handling and cooking of meat and meat products should also apply to raw fish that must always be cooked to a temperature likely to inactivate the parasite (at least 66°C) or deep frozen at −21°C for 1 or 28 days (for unsporulated or sporulated oocysts, respectively) (Dumètre & Dardé, 2003) since storing in household refrigerators (4°C for 6–11 weeks) does not prevent the development of oocysts infectivity (Lindsay, Blagburn, & Dubey, 2002).

Additional fields investigations are of course recommended to improve our understanding of the role of market fish as potential vector for *T. gondii* whereas the issue of oocysts viability remained to be assessed to unravel the parasite transmission pathways in the sea environment and to evaluate the risk of foodborne toxoplasmosis for human consumers.

## CONFLICTS OF INTERESTS

The authors declared no potential conflicts of interest with respect to the research, authorship and/or publication of this article.
